# Challenges in establishing animal models for studying osteoimmunology of hypoparathyroidism

**DOI:** 10.3389/fvets.2023.1163903

**Published:** 2023-04-26

**Authors:** Maria Butylina, Ursula Föger-Samwald, Katharina Gelles, Peter Pietschmann, Wolfgang Sipos

**Affiliations:** ^1^Institute of Pathophysiology and Allergy Research, Center for Pathophysiology, Infectiology and Immunology, Medical University of Vienna, Vienna, Austria; ^2^Clinical Department for Farm Animals, University of Veterinary Medicine Vienna, Vienna, Austria

**Keywords:** animal models, bone, PTH, T cells, interleukins, TNF-α

## Abstract

Hypoparathyroidism is a relatively rare human and veterinary disease characterized by deficient or absent production of parathyroid hormone (PTH). PTH is known as a classical regulator of calcium and phosphorus homeostasis. Nevertheless, the hormone also appears to modulate immune functions. For example, increased CD4:CD8 T-cell ratios and elevated interleukin (IL)-6 and IL-17A levels were observed in patients with hyperparathyroidism, whereas gene expression of tumor necrosis factor-α (TNF-α) and granulocyte macrophage-colony stimulating factor (GM-CSF) was decreased in patients with chronic postsurgical hypoparathyroidism. Various immune cell populations are affected differently. So, there is a need for validated animal models for the further characterization of this disease for identifying targeted immune-modulatory therapies. In addition to genetically modified mouse models of hypoparathyroidism, there are surgical rodent models. Parathyroidectomy (PTX) can be well performed in rats—for pharmacological and associated osteoimmunological research and bone mechanical studies, a large animal model could be preferable, however. A major drawback for successfully performing total PTX in large animal species (pigs and sheep) is the presence of accessory glands, thus demanding to develop new approaches for real-time detection of all parathyroid tissues.

## 1. Parathyroid glands and parathyroid hormone—an introduction

**Figure 1:**
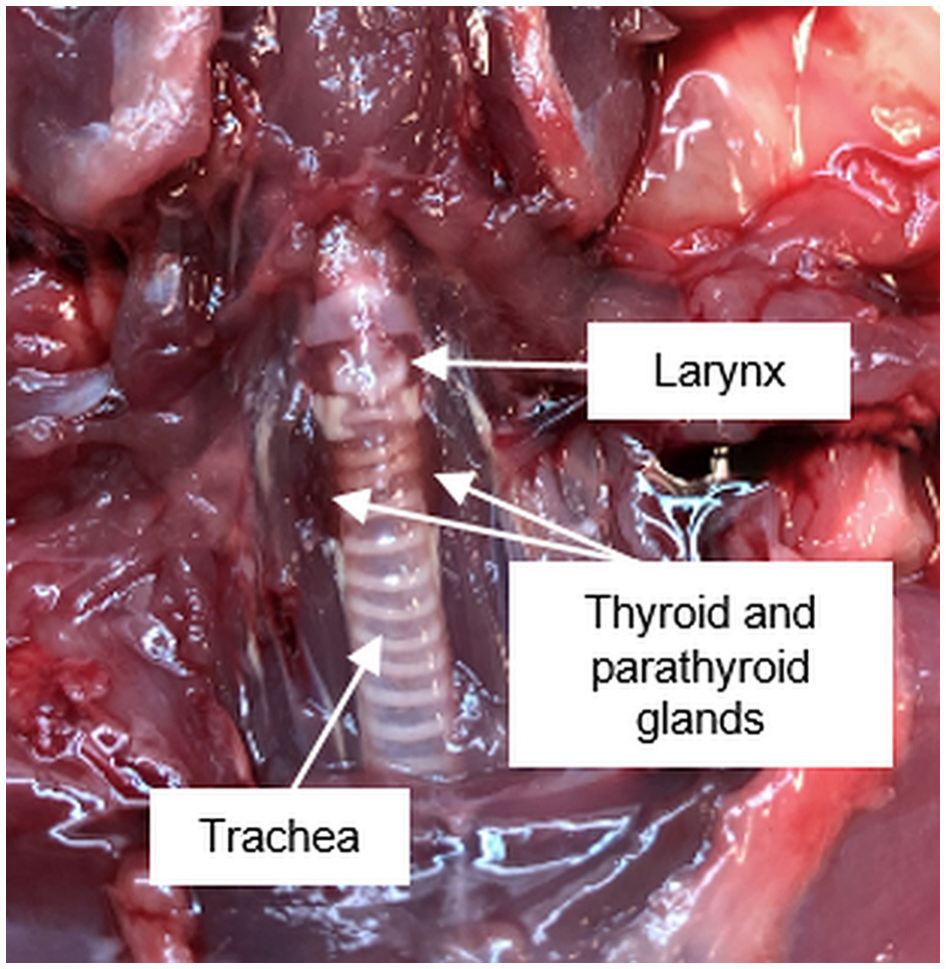
Anatomical position of the pharyngeal and ventral neck region of a Sprague Dawley rat.

**Figure 2:**
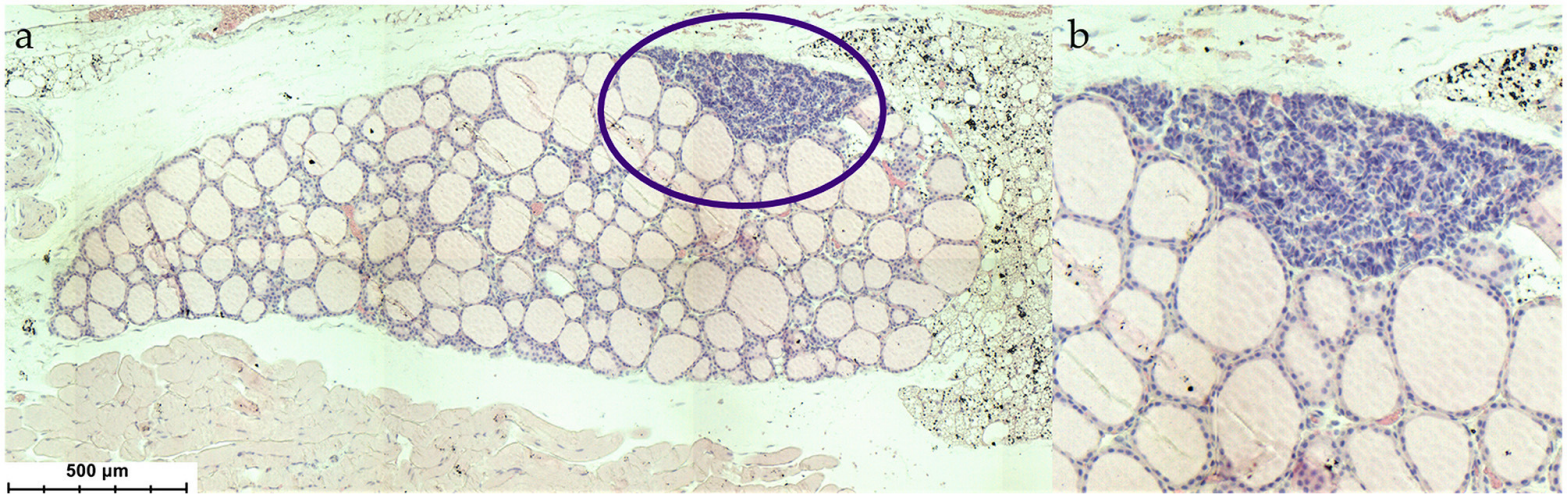
**(a)** Histological situs of the parathyroid gland within the thyroid (*Mus musculus*), **(b)** magnification of the parathyroid gland. HE stain.

The first description of the parathyroid gland is credited to Sir Richard Owen, who in 1862 published the findings of the autopsy of an Indian Rhinoceros (*Rhinoceros unicornis*) he dissected in the winter months of 1849/1850 (1). The term “parathyroid gland” was coined by Ivar Viktor Sandström, who in 1877 identified the gland in a dog and subsequently in cats, rabbits, horses, and humans (2). The pathologist Jakob Erdheim proved in a series of experiments with rats that total parathyroidectomy leads to tetany and for the first time related the parathyroid gland to calcium metabolism (3). The parathyroid glands are important organs, which are located in the neck posterior and inferior to the thyroid gland and emerge from the third and fourth pharyngeal pouches (Figures 1, 2).

In humans, there are usually four glands, which are crucial for the maintenance of blood calcium homeostasis, as parathyroid glands are responsible for parathyroid hormone (PTH) secretion. Furthermore, lower levels of PTH transcripts can also be determined in the thymus, pituitary, and hypothalamus (4). PTH is located in the secretory granules and is secreted from the chief cells in response to reduced circulating ionized calcium concentrations to maintain the normocalcemic state. Usually, a minimal proliferation of the parathyroid cells can be seen, but chronic hypocalcemia triggers an increase in size and number of the parathyroid cells (5). On the plasma membrane of the parathyroid cells, calcium-sensing receptors (CaSRs) are abundantly present, which are responsible for monitoring free calcium concentrations and binding Ca^2+^ (6–12). The CaSR belongs to the G-protein-coupled receptor superfamily, which has a calcium-binding element in the extracellular domain and signaling determinants in the cytoplasmic region (13). Outside the parathyroid gland, CaSRs play a crucial role in the kidneys in taking part in the regulation of urinary calcium excretion independently of PTH (14). Furthermore, CaSRs can be found in the intestine, vasculature, and lungs (13).

PTH is a peptide hormone consisting of 84 amino acids and is—together with vitamin D—essential for blood calcium homeostasis. It belongs, together with PTH-related peptide (PTHrP) and tuberoinfundibular peptide of 39 residues (TIP39), to the parathyroid hormone peptide subfamily (4). Additionally, a new member was discovered, PTH-like peptide (PTH-L), which is only existing in non-mammalian species like teleost fishes, chicken, or *Xenopus* (15). In mammals, PTH is first synthesized as a pre-pro-peptide consisting of 115 amino acids, but only the 84 amino acids full-length single-chain polypeptide is later secreted by the parathyroid glands (9). It is responsible for inducing the release of calcium and phosphate from the skeletal reservoir by bone resorption while simultaneously acting on the kidneys. High levels of extracellular calcium inhibit PTH secretion, while low serum calcium levels lead to an increase, which has an effect on the PTH receptor in the kidneys leading to higher resorption of tubular calcium and suppression of phosphate reabsorption (12, 13). Persistently decreased systemic calcium levels further lead to an upregulated PTH mRNA expression and an increased number of PTH-secreting parathyroid cells (16). As a consequence, there is a rise in the renal production of 1,25(OH)_2_VitD_3_, leading to enhanced intestinal calcium absorption, which is a negative regulator of PTH secretion (6, 12, 17). Decreased levels of VitD_3_ in turn lead to higher PTH production (18). Furthermore, PTH indirectly activates bone-resorbing cells, the osteoclasts, through the classical parathyroid hormone 1 receptor (PTH1R). This receptor is expressed mainly on osteoblasts and in the kidneys. Activated osteoblasts then activate osteoclasts *via* the RANKL (receptor activator of nuclear factor-κB ligand)–RANK axis, which results in increased net bone resorption (12, 19).

## 2. Pathophysiology and clinical aspects of hypoparathyroidism in humans

Hypoparathyroidism is a relatively rare disease characterized by deficient or absent production of PTH (with blood-serum PTH levels below the physiological reference range of 12–72 ng/L corresponding to 1.5–6.0 pmol/L), which leads to a disbalanced extracellular fluid calcium level. Low calcium levels can either have rapid onset or successively develop almost asymptomatically. On the contrary, serum phosphate levels typically are increased. In consequence, the calcium/phosphate ratio is decreased (but increased in primary hyperparathyroidism). The majority of human cases of hypoparathyroidism results from neck—in particular thyroid—surgery (20–22).

In addition to hypoparathyroidism resulting after neck surgery, non-surgical or genetic forms of hypoparathyroidism can be described. The most frequent genetic form is the DiGeorge syndrome, which affects approximately 60 % of children diagnosed with hypoparathyroidism (23). It emerges from a microdeletion in chromosome 22q11.2, leading to a lack of T box protein 1, which is crucial for the development of the thymus and parathyroid glands. Due to this deletion, cardiovascular malformations, thymus underdevelopment, and facial abnormalities arise (24, 25). Furthermore, affected children show symptoms like chronic infections, nasal regurgitation, hypocalcemia, feeding difficulties, and learning disabilities (26).

Another genetic cause for hypoparathyroidism is the autoimmune polyendocrine syndrome type 1 (APS-1), which is described as an autosomal recessive disorder and caused by a mutation in the autoimmune regulator gene *AIRE* on chromosome 21q22.3 (27, 28). This mutation gives rise to a lack of self-immunotolerance, leading to the destruction of the parathyroid, adrenal, and other endocrine glands. APS-1 usually develops during early childhood at an age of 2–5 years (29). For the diagnosis of autoimmune polyendocrine syndrome type 1, two of three major diseases must be present, including hypoparathyroidism, adrenal insufficiency, and mucocutaneous candidiasis (30). These diagnostic criteria are often met before an age of 20 years (31).

Typical symptoms occurring during hypoparathyroidism, often due to low calcium levels, are, tetanic spasms, which may be lethal (20). Regarding bone manifestations, affected persons show higher bone mineral densities than sex- and age-matched controls (32). Patients with hypoparathyroidism exhibit changes in bone metabolism, as low-normal values of bone turnover markers were detected in blood and urine (33). Consequently, hypoparathyroidism leads to a greater risk of developing fractures in the appendicular skeleton (34). Affected patients often show neuromotor manifestations, like, for example, that of parkinsonism, which partially improved after the treatment of hypocalcemia. Some patients also display increased anxiety, fatigue, difficulty to concentrate, and a decrease in memory (20). Furthermore, hypoparathyroidism is associated with heart failure with resistance to diuretics and other standard treatment options. Patients with acute hypocalcemia may also show hypotension, bradycardia, and arrhythmias. After the correction of hypocalcemia, patients with cardiac dysfunction showed improvement (35, 36). Regarding the gastrointestinal tract, patients often exhibit abdominal cramps and constipation (20). Moreover, hypoparathyroidism is often associated with intense photophobia, chronic conjunctivitis, and cataracts (37). Regarding cutaneous manifestations, patients suffer from dry skin, pustular psoriasis, or deformations of the nails (38). The most common dental manifestation of hypoparathyroidism is hypoplastic enamel followed by cemental hyperplasia (39).

To correct hypocalcemia, patients with hypoparathyroidism are treated with calcium supplements and vitamin D analogs (40). Nevertheless, despite (standard) treatment, the quality of life of many patients is impaired due to hypoparathyroidism-associated symptoms (41, 42). Recently, a novel treatment option with human recombinant PTH has become available (43–45) and has been associated with an improved quality of life (46). Probably due to the rarity of the disease, rigorous data on co-morbidities of hypoparathyroidism are relatively sparse. Patients with postsurgical hypoparathyroidism are at an increased risk of renal complications and hospitalization due to seizures (47). In a study based on the Danish National Patient Registry, Underbjerg and co-workers demonstrated that postsurgical hypoparathyroidism is associated with a significantly increased risk of hospitalization for infections (and depression/bipolar disorders) (48). This increased risk might be due to a compromised immune function (49). Interestingly, when patients with urinary tract infections (potentially resulting from urinary calcium deposition) were excluded from the analyses, the increased risk of hospitalization for infections persisted. In interpreting their results, the authors assumed that “PTH may impair the immunology response to infections” (47). In 2015, these authors extended their observation of an increased risk of hospitalization due to infections also to patients with non-surgical hypoparathyroidism (34). In a subsequent study, Underbjerg et al. investigated potential biochemical risk factors associated with infections. Persistent hyperphosphatemia, which develops due to a diminished renal excretion in hypoparathyroidism, was associated with increased mortality and risk of any infections (50).

## 3. Osteoimmunology of hypoparathyroidism

**Figure 3:**
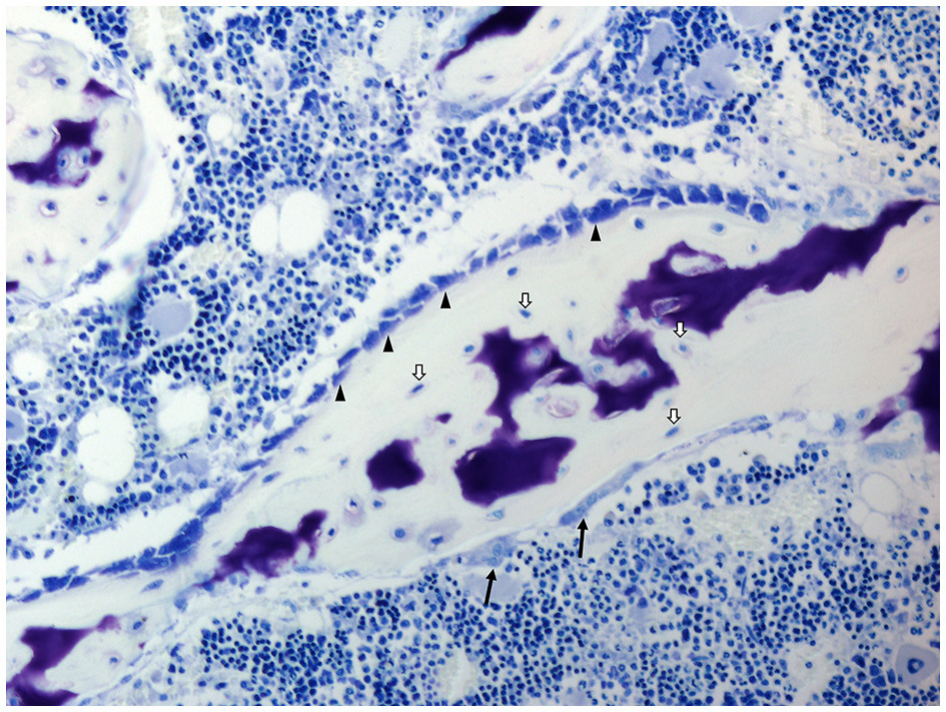
Histology of the bone of a domestic pig; toluidine blue stain. Osteoblasts (arrowheads), osteocytes (white arrows), and osteoclasts (black arrows). Cell types are identified due to their morphological characteristics and position within specific bone compartments. Porcine bone marrow-derived stromal cells as well as osteoblasts and osteoclasts exhibit similar *in vitro* growth characteristics and osteoimmunological properties as their rodent and human counterparts (51, 52).

Osteoimmunology analyses the interplay between bone and immune cells. The main two types of bone cells are osteoblasts and osteoclasts. Osteoblasts are the so-called bone-forming cells, which are responsible for bone matrix formation and mineralization, whereas bone resorption is performed by osteoclasts. In addition to osteoblasts and osteoclasts, osteocytes and bone lining cells can be found in bones (Figure 3).

Mature osteoblasts are the only cell type that is able to construct bones by secreting bone matrix proteins and guidance of mineralization. Bone-forming cells are cuboidal cells, which are present along the bone surface covering 4–6% of the total resident bone cells. Although mature osteoblasts are short-lived, a subset is differentiating into osteocytes that are encapsulated within the newly formed bone matrix. Osteocytes are characterized as the most abundant cell type (90%) present within the matrix or on bone surfaces, where they are responsible for supporting bone structure and metabolism. Furthermore, they are in charge of mechanosensation as they transduce stress signals from bending or stretching bone into biological activity. Those, which are not encapsulated, undergo apoptosis or become inactive flat-shaped bone lining cells, which cover the bone surface, where neither bone resorption nor formation occurs in humans. Bone lining cells prevent bone resorption by blocking the interaction between osteoclasts and the bone matrix, which should not be degraded. Another important function is the production of osteoprotegerin (OPG) and RANKL, which are crucial for osteoclast differentiation. Bone-resorbing osteoclasts are large multinucleated cells, which origin from the hematopoietic lineage. During resorption, osteoclasts secrete tartrate-resistant acid phosphatase (TRAP), cathepsin K, hydrogen ions, matrix metalloproteinase-9, and gelatinase, which are necessary for the digestion of the organic matrix. Dysregulations in the activity of osteoblasts can lead either to an increased or reduced bone mass (17, 53–56).

As mentioned before, PTH is a classical regulator of calcium and phosphorus homeostasis. Nevertheless, the hormone also appears to modulate immune functions. For instance, PTH receptors were found to be expressed by cells of the innate and acquired immune system [for review, see Geara et al. (57)]. Kotzmann et al. (58) described an increased CD4:CD8 T-cell ratio in patients with primary hyperparathyroidism, characterized by increased serum interleukin (IL)-6 and IL-17A levels (59, 60), and in mice, PTH augmented the production of tumor necrosis factor-α (TNF-α) by T cells. Moreover, in a recent study aiming at evaluating the immune function in patients with chronic postsurgical hypoparathyroidism, immune cell profiling revealed a decline in different immune cell populations including monocytes and regulatory, naïve, and total CD4^+^ lymphocytes. In addition, TNF-α and GM-CSF gene expression and circulating TNF-α levels were shown to be decreased in patients with chronic postsurgical hypoparathyroidism, whereas absolute numbers of total CD3^−^CD56^+^ natural killer cells were significantly increased (49). Collectively, these findings indicate that PTH induces proinflammatory cytokines and also nominates PTH as a regulator of the crosstalk between bone and the immune system, a field termed “osteoimmunology” as mentioned before (61, 62). Regarding this crosstalk, in addition to osteoclasts, also osteocytes are assumed to play an important role. In addition to their classical role as mechanosensors, osteocytes express several central regulators of bone and mineral metabolism and therefore can be regarded as endocrine cells (63). Osteocytes regulate bone formation by the expression of the Wnt antagonist sclerostin and dickkopf-1 (DKK-1), as well as osteopontin, a negative regulator of bone mineralization; fibroblast growth factor-23 (FGF-23) is an endocrine product of osteocytes that regulates phosphate homeostasis and 1,25(OH)_2_VitD_3_ synthesis (63–65). Osteocytes also express factors that determine osteoclast generation, namely RANKL, OPG, and proinflammatory cytokines such as IL-6, IL-17, and TNF-α (66, 67).

Responsiveness of osteocytes to PTH is well established in intermittent PTH administration causing net bone formation. This osteoanabolic effect is in part caused by decreasing sclerostin (68, 69). Given the well-established crosstalk between PTH and osteocytes on the one hand and the fact that osteocytes act as regulatory cells producing proinflammatory cytokines, among other substances, on the other hand, we expect that decreased PTH levels in hypoparathyroidism lead to decreased levels of proinflammatory cytokines produced by osteocytes, thereby contributing to bone effects seen in conjunction with hypoparathyroidism such as increased bone mineral density due to low bone turnover. Due to their inaccessible location, osteocytes are a challenging cell population to study; nevertheless, a number of osteocyte cell lines are available and have facilitated their investigation *in vitro* [for review, see Dallas et al. (70)]. As an alternative approach, for *ex vivo* studies, the advantage could be taken from the fact that osteocytes are by far the most abundant cell population in bone. In a recent publication, the protein expression of osteocytes in two different mouse strains was investigated immunohistochemically. It was evident that osteocytes express important proteins, such as sclerostin, DKK-1, or periostin, which are associated with bone formation (71).

In the genetic form of hypoparathyroidism (APS-1, see above), a mutation in the *AIRE* gene causes an impaired formation of the autoimmune regulator protein. The lack of this protein is associated with the decrease in autoantigen expression in the thymus and disruption of the negative selection of T-lymphocytes (28, 30). Many affected patients show autoantibodies against interferon (IFN)-α, IFN-ω, and IL-22 (72). As stated above, *AIRE* is known to play a crucial role in the induction of T-cell tolerance (73). 75 % of patients affected with DiGeorge syndrome, another form of genetic hypoparathyroidism, show immunodeficiencies including thymic hypoplasia and an impaired T-cell production, leading to a low T-cell count (26, 74).

## 4. Animal models of hypoparathyroidism

For the establishment of a valid biomedical model, it is of interest whether the targeted pathology occurs naturally in the respective species. Hypoparathyroidism, however, has only minor significance for veterinary species, and if yes, only pet animals are worth mentioning. There are descriptions of feline primary hypoparathyroidism (75), which resulted in a reversible myocardial failure due to excessive hypocalcemia in a patient (76) as well as primary hypocalcemia in dogs, which can be treated successfully with calcium and VitD_3_ supplementation (77). Whereas there exists a series of further case reports on canine and feline disease, there are only very few documentations on (primary) hypoparathyroidism in horses, which has a genetic background in this species (78), and a single report on a case of bovine disease (79). Remarkably, in pigs and sheep, there is no single report in the literature concerning hypoparathyroidism, which is in line with the findings of accessory parathyroid glands in these species. Interestingly, even in older pigs, such as minipigs, endocrinopathies (except for sexual ones) are nearly not encountered in the veterinary practice at all (80). No records on non-mammalian tetrapod hypoparathyroidism could be found.

Cats and dogs, although principally suited for parathyroidectomy (PTX) to induce hypoparathyroid conditions, have to be excluded in Austria due to legal regulations. Hence, large animal species worth considering as models are sheep and pigs. They are comparably easy to handle and inexpensive in housing. Moreover, they are frequently used in osteologic and osteoimmunological studies as they show anatomical and physiological similarities with humans in various organs including the bone compartment (81–83). Literature research and cadaveric feasibility studies regarding parathyroid gland anatomy and topography revealed that in sheep the superior parathyroid glands are easy to detect. However, the inferior parathyroid glands are deeply embedded in the thyroidal tissue and cannot be separated from the surroundings due to a missing encapsulation. Additionally, accessory parathyroid glands are disseminated over a large area of the ventral neck and total PTX is therefore impossible. This is underlined by reports in older literature of good tolerability of PTX in sheep, which points toward only a partial hypoparathyroid condition after surgery (84).

In swine, total PTX is difficult due to accessory glands, which can functionally replace primary parathyroid tissue. Moreover, the superior parathyroid glands are found in variable numbers and at variable sites. They are localized deeply in the massive cranio-ventral neck region in near vicinity to vulnerable structures and can easily be confused with other organs such as lymphatic tissue or thymus lobules. In recent literature, there is one report describing a new approach for PTX surgery in swine, however, without reporting on the effects of PTX (85). Another group described the effects of thyroparathyroidectomy (TPTX), but not isolated PTX, on bone development in unborn sheep (86). Taken together, both sheep and swine do not fulfill anatomical requirements for a suitable conventional surgical hypoparathyroidism model. Instead, these species ask for novel ways to visualize dispersed parathyroid tissues when choosing a surgical approach.

Existing genetically modified animal models of hypoparathyroidism include mice carrying parathyroid hormone (PTH)-null (87) or Glial Cells Missing Homolog 2 (GCM2)-null alleles (88). Whereas in the first model PTH levels are decreased by directly targeting the PTH gene, in the second model parathyroid gland development is impaired by targeting a transcription factor crucial for gland development. Unexpectedly, GCM2-deficient mice, despite their lack of parathyroid glands, displayed only a mildly abnormal bone phenotype with PTH levels that were identical to those in wild-type mice. Further studies revealed the thymus as an additional source of PTH compensating for impaired PTH output of the parathyroid gland (88). The use of these models for acquired hypoparathyroidism—the most common form of the disease (89)—is limited by an inherited chronic hypoparathyroid phenotype. Moreover, alterations in the development of organs affected by impairments of the hypothyroid gland (i.e., the skeleton) may occur. To overcome this limitation, Bi et al. established the PTHcre-iDTR mouse model, in which parathyroid cells selectively express the human diphtheria toxin receptors (DTR) (90). By systemic injection of diphtheria toxin, parathyroid cells can be ablated leading to low PTH levels and an acquired hypoparathyroid condition. There are also several genetic models mimicking related syndromes including impaired parathyroid gland development, such as the DiGeorge syndrome, the hypoparathyroidism–sensorineural deafness–renal dysplasia (HDR) syndrome, and the hypoparathyroidism–retardation–dysmorphism (HRD) syndrome [for review, see Garfield and Karaplis (91)]. Knockout mice are typically used as models, but there is also a zebra fish model mimicking the HRD syndrome (92). Given that these models failed to display conclusive symptoms of hypoparathyroidism (93–96), they are not suitable for use as models for that issue. Apart from this, in these syndromes, defects of the parathyroid gland are associated with other manifestations, making a comparison to hypoparathyroidism in humans difficult.

As an alternative to genetic models, surgical PTX is used to model acquired hypoparathyroidism. Mice, due to the small size of the parathyroid glands, are limited in their use for PTX. Hence, Bi et al. developed a mouse model in which green fluorescent protein (GFP) is selectively expressed in the parathyroid gland, thus facilitating a more precise PTX surgery (90). Another approach to overcome the limitation of small parathyroid glands in mice is the performance of TPTX (97). In this model, hormones of the thyroid gland (thyroid hormones and calcitonin) have to be supplemented, thereby reducing comparability to acquired hypoparathyroidism in humans. PTX was performed also in chickens and rabbits; however, most typically it is performed in rats (98–105). Comparable to mice, also in rats, fluorescence-based detection was used to enable precise excision of the parathyroid gland. To prevent animals from perishing, calcium must be supplemented. The ideal dietary calcium content in this model to mimic acquired hypoparathyroidism in humans was determined to be 0.5%. Serum calcium levels decreased, and phosphorus levels and bone volume increased (106, 107). Cadaveric studies performed by us revealed that rats are preferable to mice from a surgical perspective.

Thus, the seemingly most reliable model of hypoparathyroidism at the moment are Sprague Dawley rats being subject to PTX at the age of 8 weeks (106). As postsurgical hypoparathyroidism is more frequent in women than in men (54), female rats should be preferred. As an alternative for surgery, male Wistar rats treated with cinacalcet may be used for the development of a non-surgical rodent hypoparathyroid model. Cinacalcet suppresses the calcium levels, which was associated with a decline in PTH, and afterwards PTH 1-34 or a delayed-clearance PTH molecule (DC-PTH) were administered to reverse this effect (108). An advantage of this model is that there is no need to replace thyroid hormones (as in most surgical models). Nevertheless, it should be mentioned that long-term data are missing for this approach.

## 5. Future perspectives

Postsurgical hypoparathyroidism has been shown to be associated with a significantly increased risk of hospitalization due to infections. Deepening the knowledge on the osteoimmunological aspects of hypoparathyroidism will lead to a better understanding of the pathophysiological mechanisms behind this observation. In particular, the influence of decreased PTH levels on the ratio of pro- vs. anti-inflammatory cytokines and dynamics in responsible cell type populations should be assessed *in vivo* using rodent models as a first step. Moreover, by investigating the impact of PTH deficiency on the production of proinflammatory cytokines by osteocytes, a possible new role of osteocytes in linking PTH and hypoparathyroidism-related bone effects might be established. As mentioned before, lowered circulating TNF-α levels were observed in patients with chronic postsurgical hypoparathyroidism (49). We, therefore, expect not only a higher bone volume, as already described, but also a higher bone mineral content and increased cortical and trabecular thicknesses in PTX models and an altered cytokine profile of various immune cell populations and osteocytes. For future research, it would be an option to develop real-time detection methods for identifying accessory parathyroid tissue islets, thus being able to perform accurate total PTX also in large animal species, as these have advantages in further pharmacological studies.

## 6. Conclusion

Acquired hypoparathyroidism is the most common form of this rare endocrinopathy in humans with a reported range of prevalence of chronic hypoparathyroidism from 6.4 to 37/100,000 (109). Except for the PTHcre-iDTR mouse model (90), most genetic models available do not reflect acquired hypoparathyroidism satisfactorily. Moreover, concomitant alterations of immune functions that could interfere with the interpretation of results cannot be excluded, and PTHcre-iDTR mice are limited by high purchase costs. Hence, a surgical model of hypoparathyroidism is most suitable to meet clear criteria. Large animal models (sheep and pig)—although their immune system is also well characterized meanwhile (81, 110, 111), which is an argument in favor of their increased use in biomedical research in general, but also in osteological research in special (112)—do not fulfill the requirements of surgical feasibility and have thus to be excluded for such experiments till real-time detection methods for hypoparathyroid tissues exist. A model widely used for hypoparathyroidism and offering high availability of analytical reagents is PTX in mice and rats. Although the small size of mice limits the amount of tissue and biological fluids that can be harvested, PTX in rats seems to be the preferable option to date. One major limitation in all experimental models of hypoparathyroidism is the fact that over 80 % of respective human patients develop this condition as a complication of anterior neck surgery due to thyroid adenomas or related pathologies, meaning that data extrapolation of sole PTX (vs. TPTX) models needs to be done with caution.

## Author contributions

PP and WS: conceptualization. MB, PP, and WS: writing—original draft preparation. MB, UF-S, KG, PP, and WS: writing—review and editing. PP: funding acquisition. All authors have read and agreed to the published version of the manuscript.
